# Elevated Systemic Inflammation Is Associated with Reduced Corticolimbic White Matter Integrity in Depression

**DOI:** 10.3390/life12010043

**Published:** 2021-12-28

**Authors:** MacGregor Thomas, Jonathan Savitz, Ye Zhang, Kaiping Burrows, Ryan Smith, Leandra Figueroa-Hall, Rayus Kuplicki, Sahib S. Khalsa, Yasuyuki Taki, Tracy Kent Teague, Michael R. Irwin, Fang-Cheng Yeh, Martin P. Paulus, Haixia Zheng, on behalf of Tulsa 1000 Investigators

**Affiliations:** 1 Laureate Institute for Brain Research, Tulsa, OK 74136, USA; MThomas@laureateinstitute.org (M.T.); jsavitz@laureateinstitute.org (J.S.); kburrows@laureateinstitute.org (K.B.); RSmith@laureateinstitute.org (R.S.); LFigueroa-Hall@laureateinstitute.org (L.F.-H.); rkuplicki@laureateinstitute.org (R.K.); skhalsa@laureateinstitute.org (S.S.K.); mpaulus@laureateinstitute.org (M.P.P.); 2 Oxley College of Health Sciences, The University of Tulsa, Tulsa, OK 74119, USA; 3 Department of Aging Research and Geriatric Medicine, Institute of Development, Aging and Cancer, Tohoku University, Sendai 980-8575, Japan; zhang.ye.r5@dc.tohoku.ac.jp (Y.Z.); yasuyuki.taki.c7@tohoku.ac.jp (Y.T.); 4 Department of Geriatric Medicine and Neuroimaging, Tohoku University Hospital, Sendai 980-8574, Japan; 5 Smart-Aging Research Center, Tohoku University, Sendai 980-8575, Japan; 6 Department of Surgery, University of Oklahoma School of Community Medicine, Tulsa, OK 74135, USA; kent-teague@ouhsc.edu; 7 Department of Psychiatry, University of Oklahoma School of Community Medicine, Tulsa, OK 74135, USA; 8 Department of Biochemistry and Microbiology, Oklahoma State University Center for Health Sciences, Tulsa, OK 74107, USA; 9 Cousins Center for Psychoneuroimmunology at UCLA, Los Angeles, CA 90095, USA; mirwin1@ucla.edu; 10 Semel Institute for Neuroscience at UCLA, Los Angeles, CA 90024, USA; 11 David Geffen School of Medicine, Los Angeles, CA 90095, USA; 12 Department of Neurological Surgery, School of Medicine, University of Pittsburgh, Pittsburgh, PA 15260, USA; frank.yeh@pitt.edu

**Keywords:** CRP, MDD, white matter integrity, quantitative anisotropy, connectometry analysis, diffusion MRI

## Abstract

(1) Background: Growing evidence indicates that inflammation can induce neural circuit dysfunction and plays a vital role in the pathogenesis of major depressive disorder (MDD). Nevertheless, whether inflammation affects the integrity of white matter pathways is only beginning to be explored. (2) Methods: We computed quantitative anisotropy (QA) from diffusion magnetic resonance imaging as an index of white matter integrity and regressed QA on C-reactive protein (CRP), controlling for age, sex, and BMI, in 176 participants with MDD. (3) Results: The QA values of several white matter tracts were negatively correlated with CRP concentration (standardized beta coefficient = −0.22, 95%CI = −0.38–−0.06, FDR < 0.05). These tracts included the bilateral cortico-striatal tracts, thalamic radiations, inferior longitudinal fasciculi, corpus callosum (the forceps minor portion and the tapetum portion), cingulum bundles, and the left superior longitudinal fasciculus III. Importantly, the association remained robust after regressing up to twelve potential confounders. The bilateral fornix and a small portion of the thalamic radiation showed a positive association with CRP levels, but these associations did not remain significant after adjusting for confounders. (4) Conclusions: Peripheral inflammation may contribute to the etiology of MDD by impacting the microstructural integrity of brain corticolimbic white matter pathways.

## 1. Introduction

An increasing number of human and animal studies indicate that systemic inflammation can induce corticolimbic circuit dysfunction and may contribute to the onset and maintenance of major depressive disorder (MDD) [1,2,3,4,5,6]. Neuroimaging studies have characterized a number of cortical and sub-cortical brain regions that are particularly sensitive to inflammation, including the amygdala, ventral striatum, insula, cingulate cortex, orbitofrontal cortex, and hippocampus [4,5,6]. Previous studies have suggested that structural and functional abnormalities in these regions may underlie some of the phenotypic traits of depression, including anhedonia, emotion dysregulation, cognitive impairment, and anxiety [3,7,8,9]. Interestingly, recent network-based analyses have also shown that reduced resting-state functional connectivity within and between these corticolimbic regions is associated with elevated markers of inflammation [4,10,11,12]. Given the fact that white matter pathways support the function and functional connectivity of the aforementioned brain regions [13,14], systemic inflammation may alter structural connections between these regions, leading to functional alterations. Nevertheless, the relationship between systemic inflammation and the integrity of white matter pathways is only beginning to be explored. The insights into this association should help to advance the mechanistic understanding of the interplay between peripheral inflammation and the structural and functional brain alterations observed in MDD.

Peripheral blood C-reactive protein (CRP) is commonly used in clinical practice as a marker for systemic inflammation. CRP is synthesized by the liver and is regulated as part of the human acute-phase innate immune response [15,16]. CRP is induced by interleukin (IL)-6 and is moderately correlated with the concentrations of IL-6 and other inflammatory cytokines such as interleukin 1 beta (IL-1β) and tumor necrosis factor (TNF) [17,18]. Importantly, a previous study also demonstrated that plasma CRP is strongly correlated with cerebrospinal fluid CRP (*r* = 0.855) [18]. In the context of MDD, CRP is among the most replicable inflammatory markers to be associated with MDD, as no less than five meta-analyses have reported that CRP is elevated in patients with MDD [19,20,21,22,23]. Furthermore, prospective data from longitudinal studies suggest that higher CRP is associated with an increased risk of depressive symptom development [22,24]. Thus, CRP is a well-established inflammatory marker to explore the relationship between systemic inflammation and white matter’s microstructural integrity in the context of MDD.

The neuroinflammation hypothesis of depression suggests that systemic inflammation can attenuate brain structural integrity via immune-mediated neurotoxic effects [1,2,3,4,25,26]. To the best of our knowledge, there are only three previous studies directly testing the association between peripheral inflammatory markers and white matter integrity in MDD, as indexed by reduced fractional anisotropy (FA) values. One study compared 35 first depressive episode and drug-naïve MDD patients with 35 age- and sex-matched healthy controls, and found that IL-1β was inversely correlated with FA values in the bilateral inferior fronto-occipital fasciculus, left uncinate fasciculus, and genu of the corpus collosum in the MDD group [27]. Another study compared 22 patients with MDD and 22 healthy subjects. They reported similar findings, showing that peripheral concentrations of TNF were negatively correlated with FA in several white matter regions, including the corpus callosum and both the left anterior and superior corona radiata [28]. In one study that pooled MDD and healthy control participants (*n* = 590), the results indicate that methylation-based measures of CRP were associated with widespread reductions in FA, and the strongest relationships were found in the external capsule and the anterior limb of the internal capsule [29]. However, all three of these studies used a tract-based spatial statistics (TBSS) approach, which projects volumetric data onto a white matter skeleton to gain statistical power through dimensionality reduction [30]. Although TBSS is a popular approach for voxel-based analysis of diffusion tensor imaging data, weaknesses in this approach have been highlighted in relation to its fully automatized analysis method [31,32,33,34]. One major concern about TBSS is that the skeleton projection step is largely biased in anatomical specificity and accuracy because the skeletonization reduces white matter tracts to a one voxel-thick sheet [34]. Therefore, interpreting results obtained from TBSS can be challenging and may not necessarily reflect pathology. Thus, while previous studies have provided some evidence to support the hypothesis that CRP levels affect white matter integrity in MDD, the anatomical specificity of this effect remains unclear.

Here, we hypothesized that individuals with high CRP levels, indicating an ongoing inflammatory process, relative to those with low CRP levels, show reduced white matter tract integrity, particularly among brain regions which have been shown previously to be sensitive to peripheral inflammatory processes. To test this hypothesis, we used a novel connectometry analysis [35] that can identity differences with high spatial specificity and comprehensively map the white matter pathways affected by systemic inflammation in patients with MDD. We further explored the association between identified white matter tracts and depressive symptoms.

## 2. Materials and Methods

### 2.1. Participants

Approval for the study was obtained from the Western Institutional Review Board (#20101611), and written informed consent was obtained from all participants. The current study included 176 patients aged 18–55 years who received a DSM-5 diagnosis of MDD (with or without comorbid anxiety). All participants were evaluated in person with the Mini International Neuropsychiatric Inventory (MINI) [36] with well-trained psychiatric clinical interviewers. Participants were drawn from the first half of the Tulsa 1000 (T1000) study (the second half of samples are currently not available) [37]. Data were collected between January 2015 and February 2017. Participants were recruited from the Laureate Psychiatric Clinic and Hospital, other local behavioral and mental health providers, and through newspaper, flyer, online, radio, and other media advertisements in the Tulsa metropolitan area. Exclusion criteria included comorbid psychiatric disorders (except for anxiety disorders), substance use disorders, neurological disorders, significant or unstable medical conditions (including cardiac vascular, gastrointestinal, endocrine, neurological hematological, rheumatological or metabolic disorders), a history of moderate-to-severe traumatic brain injury, a history of autoimmune disorders (except hypothyroidism), a positive urine drug screen, a body mass index (BMI) <17 or >38 kg/m^2^, and general MRI exclusion criteria (details in [37]).

### 2.2. Behavioral Data

Participants completed the Patient-Reported Outcomes Measurement Information System (PROMIS) [38] scales for depression and anxiety, Patient Health Questionnaire 9 (PHQ-9) [39] for depressive symptoms, the Customary Drinking and Drug Use Record (CDDR) structured interview for lifetime alcohol use [40], as well as the childhood trauma questionnaire (CTQ) for early life stress [41]. Participant demographic and behavioral data are summarized in Table 1.

### 2.3. C-Reactive Protein

Morning blood samples were used to isolate serum following standard laboratory procedures and stored at −80 °C. Serum concentrations of C-reactive protein (CRP) were analyzed in duplicate with V-PLEX Neuroinflammation Panel-1 Human Kits and a Meso Quickplex SQ120 instrument (Meso Scale Diagnostics, Rockville, MD, USA). The lowest level of quantification (LLOQ) was 0.027 mg/L, and the intra- and inter-assay coefficients of variation were 2.34% and 10.04%, respectively. CRP concentration was log-transformed, and outliers (defined as an absolute value larger than three standard deviations from the mean) were removed from analyses.

### 2.4. MRI Data Acquisition and Preprocessing

Diffusion MRI scans were acquired using two identical 3.0T scanners (GE Discovery MR750) with brain-dedicated receive-only 32 element coil arrays optimized for parallel imaging (Nova Medical, Inc., Wilmington, MA, USA). The diffusion-weighted imaging (DWI) data were acquired using a single-shell acquisition with 60 diffusion encoding directions (*b* value = 1000 s/mm^2^, TR/ TE = 9000/83.6 ms, with acquisition and reconstruction matrix = 128 × 128, field of view (FOV) = 25.6 × 25.6 cm, slice thickness = 2 mm, without interslice spacing, 73 axial slices, acceleration factor R = 2 in the phase encoding direction) and 8 no diffusion-weighted images (*b* value = 0 s/mm^2^) acquired at the beginning of the scan. The total acquisition time was 10 min and 50 s.

DWI data were preprocessed using the FMRIB Software Library tool (FSL, version 6.0, https://fsl.fmrib.ox.ac.uk/fsl, accessed on 30 August 2021) and the DSI Studio (30 August 2021 build, http://dsi-studio.labsolver.org). The FSL ‘eddy’ tool was used to estimate and correct eddy current-induced distortions and gross participant movement [42]. The quality of the dataset was assessed using the eddy QC tools [43]. Slices with signal loss caused by participant movement coinciding with the diffusion encoding were detected and replaced by predictions made by means of a Gaussian process [44]. The quality control criteria were set as an average absolute volume to volume head motion of <3 mm, or total outliers <5%. Skull stripping was performed for each participant using FSL-BET [45]. The diffusion data were reconstructed in the MNI space using q-space diffeomorphic reconstruction to obtain the spin distribution function (SDF) with the default settings of DSI Studio. A diffusion sampling length ratio of 1.25 was used, and the output resolution was 2 mm [46,47]. The reconstructed results of each subject were also inspected. Although fractional anisotropy (FA) is a commonly used white matter integrity index, it has been shown that FA is susceptible to partial volume effects and crossing fiber issues, which limit the fiber tracking accuracy [48,49,50,51]. In comparison, a previous study demonstrated that quantitative anisotropy (QA) is highly correlated with FA and less sensitive to the partial volume effects of crossing fibers and free water [52]. Thus, QA maps were computed for each subject using DSI Studio and served as a white matter integrity index in the following analyses.

### 2.5. Connectometry Analysis

Connectometry is a novel diffusion MRI analytic approach that utilizes a multivariable linear regression model and permutation testing to identify the subcomponents of the white matter tracts that show association with a variable of interest [35]. In the current study, diffusion MRI connectometry analyses were performed using DSI Studio to map the specific white matter pathways correlated with CRP concentration. A multiple regression model was used to identify the association between CRP concentration and QA value at each voxel level, controlling for age, sex, and BMI. Local voxels exceeding a t-statistic threshold of two for a CRP effect on QA were selected, and fiber tracking was performed via a deterministic fiber tracking algorithm [52]. This deterministic fiber tracking algorithm allows for crossing fibers within voxels, which helps to reduce false-positive connections and ensure an excellent valid connection rate (i.e., 92%) [53,54]. Track trimming was set with a default value of one iteration. A length threshold of 20 voxels was used to identify associated white matter tracts. Bootstrap resampling with 2000 randomized permutations was used to estimate the null distribution of track length and provide false discovery rates (FDR). The cerebellum was masked out from the analyses to avoid spurious findings due to partial scan coverage of the cerebellum. For more detailed methodology documentation, please see Yeh et al. (2016) [35].

### 2.6. Exploratory Analysis and Sensitivity Analysis

Once the tracts that showed a positive or negative association with CRP concentration were identified, the mean QA value was extracted to estimate the effect size and perform the following analyses. First, to explore the association between the mean QA from the identified tracts and depressive symptom severity, a linear regression model with age, sex, and BMI as covariates and depression scores was performed. The main outcome was the total PHQ-9 score, but in exploratory analyses, we also measured each of the nine PHQ-9 items individually. Second, to examine the robustness of the association between CRP concentration and QA, twelve variables that could theoretically influence the CRP concentration or cause white matter structure change, or both, were selected as potential confounders (also known as principles of confounder selection) [55]. The twelve selected variables included age, sex, BMI, education, income, early-life stress (total CTQ score), medication status (defined as whether subjects were taking psychotropic medication), current smoker, the severity of current symptoms of depression and anxiety (measured by PROMIS scales), number of episodes (obtained from MINI interview), and the lifetime alcohol use (obtained from CDDR interview). A multi-regression model was used to test whether the association between CRP and QA would be sensitive to these confounders by adding these variables in the regression model. The mean QA value was first extracted from tracts showing a negative or positive association with CRP separately. Then, two regression models were carried out, respectively.

## 3. Results

### 3.1. Tracts Correlated with CRP Concentration

**Figure 1:**
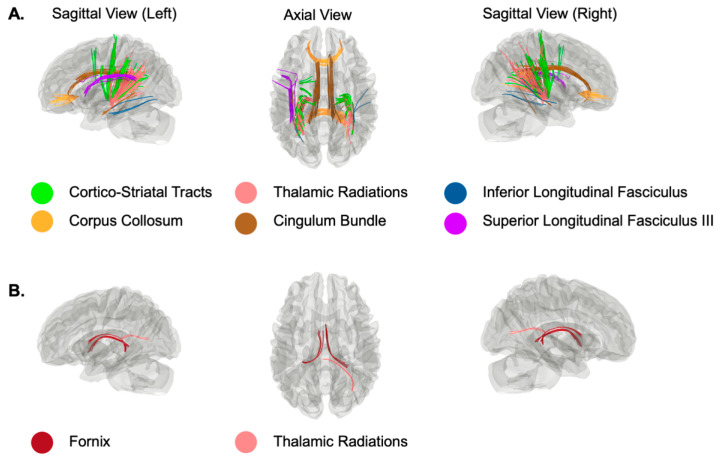
White matter tracts with QA values significantly correlated with log-transformed CRP value. (**A**) Tracts showing a negative association with log-transformed CRP value. (**B**) Tracts showing a positive association with log-transformed CRP value.

**Figure 2:**
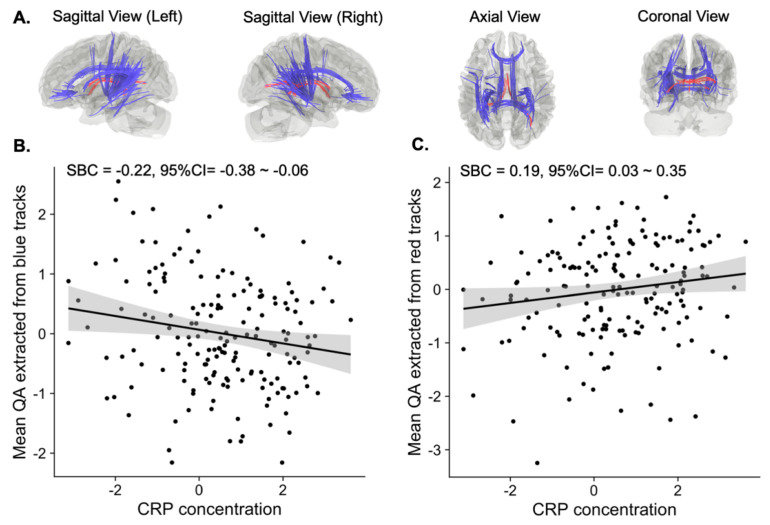
White matter tracts positively and negatively associated with the CRP concentration. (**A**) Illustration of white matter tracts positively (red) and negatively (blue) associated with the CRP concentration. (**B**) Scatter plot of mean QA extracted from the negatively associated tracts (blue tracts) and the CRP concentration. (**C**) Scatter plot of mean QA extracted from the positively associated tracts (red tracts) and the CRP concentration. The mean QA value in (**B**,**C**) regressed out age, sex, and BMI.

As shown in Figure 1A, connectometry analysis revealed several white matter tracts whose QA value was negatively correlated with CRP concentration (FDR < 0.05). Specifically, individuals with high levels of CRP (log-transformed CRP value was used) relative to those with low levels showed attenuated connectivity among bilateral cortico-striatal tracts, thalamic radiations, inferior longitudinal fasciculi, corpora callosa (the forceps minor portion and the tapetum portions), cingulum bundles, and the left superior longitudinal fasciculus III. We used these tracts as a mask to extract the mean QA value to estimate the effect size by using the same linear regression model controlling for age, sex, and BMI (Figure 2A,B). The estimated standardized beta coefficient (SBC) was −0.22 with a 95% confidence interval (95% CI) = −0.38–−0.06. Thus, for every one standard deviation increase in the log-transformed CRP value, the QA in these areas (blue area in Figure 2A) decreases by 0.22 standard deviations.

The connectometry analysis also identified a few white matter tracts that showed a positive association with CRP concentration (Figure 1B). That is, individuals with high levels of CRP relative to those with low levels of CRP show increased connectivity among the bilateral fornix and right thalamic radiations. Similarly, we estimated the effect size for this positive association (Figure 2A red area and Figure 2C). The estimated SBC was 0.19, with 95%CI = 0.03–0.35. On average, if the log-transformed CRP value increases by one standard deviation from the sample mean, the QA at these areas (red area in Figure 2A) will increase by 0.19 standard deviations from the sample mean.

### 3.2. Association between QA and Depressive Symptoms

Linear regression models with age, sex, and BMI as covariates were used to explore whether there were any associations between identified white matter tracts’ QA values and depressive symptoms. There were no significant associations between QA and specific depressive symptoms (indexed by each of the PHQ-9 items and the total PHQ-9 score). The SBC, 95%CI, and *p* values are summarized in Table 2.

### 3.3. Sensitivity to Potential Confounders

The negative association between CRP concentration and the QA value remained significant after regressing up to twelve potential confounders (SBC = −0.17, 95%CI = −0.34–−0.004, *p* = 0.04). These confounders include age, sex, BMI, education, income, early-life stress, medication status, current smoker, the severity of current symptoms of depression and anxiety, the number of episodes, and the lifetime alcohol use. The mean QA along the blue tracts in Figure 2A was used as the outcome in the regression model. That is, the bilateral cortico-striatal tracts, thalamic radiations, inferior longitudinal fasciculi, corpora callosa (the forceps minor portion and the tapetum portion), cingulum bundles, and the left superior longitudinal fasciculus III. However, the positive association between CRP concentration and QA along red tracts (i.e., bilateral fornix and right thalamic radiations) no longer remained statistically significant (SBC = 0.17, 95%CI = −0.02–0.35, *p* = 0.06).

## 4. Discussion

This study aimed to better characterize the association between systemic inflammation and white matter tract integrity. We employed novel connectometry analyses to map the association between peripheral inflammation, as indexed by CRP concentration in blood, and white matter integrity, as indexed by QA values, in 176 individuals with MDD. We found that the QA values in the bilateral cortico-striatal tracts, thalamic radiations, inferior longitudinal fasciculi, corpora callosum (the forceps minor portion and the tapetum portions), cingulum bundles, and the left superior longitudinal fasciculus III showed a robust negative correlation with CRP concentration. While a causal conclusion cannot be drawn from our current cross-sectional study, our findings suggest for the first time that peripheral CRP levels are negatively associated with the integrity of white matter tracts connecting corticolimbic regions shown to be sensitive to levels of inflammation in previous studies [4,5,6].

These findings bear some similarity to results attained by other studies, but also shed new light on the relationship between systemic inflammation and white matter integrity in depression. Previous studies have reported inverse associations between proinflammatory cytokines in the periphery (i.e., IL-1β and TNF) and corpus callosum integrity (indexed by FA value) [27,28]. The corpus callosum is the most prominent forebrain commissure connecting the left and right hemispheres, and is only found in placental mammals [56,57]. It contains over 200 million axon fibers connecting large cortico-cortical as well as cortico-subcortical pathways. Thus, the corpus callosum plays a critical role in the integration of inter-hemispheric cognitive and sensory information [56,57]. Structural abnormalities of the corpus callosum have been linked to cognitive and emotional deficits in a wide range of psychiatric disorders, including MDD and suicidality [58,59], schizophrenia [60], posttraumatic stress disorder [61], bipolar disorder [62,63], and attention-deficit hyperactivity disorder [64]. Therefore, an impaired connection between hemispheres could represent a common deficit across a broad spectrum of psychiatric disorders.

Our finding that CRP was negatively associated with corpus callosum QA values is consistent with the hypothesis that systemic inflammation may contribute to the etiology of mood disorders. As mentioned above, previous studies that have attempted to address similar research questions have used TBSS to perform the analyses, limiting the anatomical sensitivity of the findings [34]. By using connectometry analysis designed to thoroughly track the subcomponents of white matter that may be influenced by study variables, our current study provides a more detailed map of the interplay between inflammation and white matter microstructural integrity. Specifically, the forceps minor portion and the tapetum portion of the corpus callosum were most strongly associated with CRP concentration. The forceps minor, also known as the anterior forceps, connects the frontal lobes and crosses the genu of the corpus callosum. Damage to this portion of the corpus callosum has been associated with depression and fatigue in multiple sclerosis [65]. The tapetum is on the splenium of the corpus callosum and is thought to be attached to the hippocampal commissure [57]. Although the specific function of the tapetum remains unclear, a smaller splenium of the corpus callosum has been found in patients with autism, attention deficit-hyperactivity disorder (ADHD), and schizophrenia [56].

We also identified some other white matter tracts showing inverse associations with CRP concentrations that have not been reported in previous studies. This included the cingulum bundle, cortico-striatal tracts, thalamic radiations, inferior longitudinal fasciculi, and superior longitudinal fasciculus. It is noteworthy that alterations in these white matter pathways have been consistently observed in patients with MDD compared to healthy subjects [62,66,67,68,69,70,71]. The cingulum bundle is a prominent white matter tract that extends through the frontal, parietal, medial, and temporal cortices, while also interconnecting the cingulate gyrus with subcortical nuclei [72]. Therefore, it is a neural pathway that is essential for executive function and emotion processing and has been strongly implicated in a wider range of neuropsychiatric disorders, including MDD [73]. The cortico-striatal tracts and thalamic radiations also contain axonal projections to various cortical and subcortical regions, including the prefrontal cortex, thalamus, caudate, ventral striatum, amygdala, and hippocampus. These regions largely overlap with cortico-striatal-pallidal-thalamic circuits, which are thought to play a crucial role in the development of MDD [8,74,75,76]. The inferior longitudinal fasciculus is a long white matter tract that connects regions of the temporal lobe to the occipital lobe, and also includes projections running to the frontal lobe and subcortical regions [77]. This white matter tract has been implicated in facial recognition, object recognition, reading comprehension, and lexical and semantic processes. Interestingly, decreased white matter integrity in the inferior longitudinal fasciculus has been found to be negatively correlated with autobiographical memory, while white matter integrity in the fornix has been found to be positively correlated with episodic memory [77]. The superior longitudinal fasciculus is a larger interhemispheric tract, and is involved in executive functioning and emotion regulation [78]. Previous studies have found that changes in white matter integrity in the superior longitudinal fasciculus were more pronounced in MDD patients with more severe symptoms, longer duration of illness, and in those not receiving treatment [79,80]. Moreover, white matter alterations in the superior longitudinal fasciculus were observed in healthy individuals with familial depression risk compared to subjects without familial risk [81]. Interestingly, in a six-year longitudinal study, faster declines in CRP concentration predicted greater FA values in the superior longitudinal fasciculus [82]. In conjunction with this previous work, our findings suggest that systemic inflammation may disrupt the communication between corticolimbic regions by reducing the integrity of white matter, contributing to the pathology of depression.

The mechanisms underlying the inverse association between CRP levels and reduced QA values remain unclear. The neuroinflammation hypothesis of depression may provide a possible explanation, which holds that increased peripheral inflammation induces an inflammatory state in the brain, causing microstructural damage in a subset of MDD patients. Animal studies and human postmortem studies have demonstrated that stress can cause blood–brain barrier (BBB) leakiness by reducing the expression of tight junction protein claudin-5, which promotes peripheral inflammatory cytokines to cross the BBB in depression [83,84]. Once peripheral cytokines enter the brain, the activity of glial cells (i.e., microglia, astrocytes, and oligodendrocyte) can be impacted, leading to axonal demyelination and neurodegeneration [85,86,87]. In particular, the oligodendrocytes, which are responsible for white matter myelination, are thought to be most vulnerable to the damaging effects of inflammatory processes [87,88,89]. In fact, significantly decreased white matter integrity and a reduced density of oligodendroglia cells are two of the most replicated findings in the postmortem MDD samples [87]. Perhaps most relevant to our discussion are findings by Myung et al. (2016), which showed that oligodendroglia abnormalities in MDD patients with suicidal ideation were correlated with lower white matter integrity in tracts connecting the prefrontal cortex with limbic nuclei [90]. While decreased white matter integrity was not correlated with suicidality in our study, we did show decreased QA values in similar tracts, suggesting a common pathology. Nevertheless, neuroinflammation as a result of increased BBB permeability could lead to a slow, degenerative attack on oligodendrocytes in white matter tracts associated with particular depressive symptoms.

Unexpectedly, we found that QA values in a small bundle of the bilateral fornix and right thalamic radiations showed a positive correlation with measured CRP concentrations. The cellular mechanisms underlying the increased QA remain unclear. Theoretically, changes in myelination, axonal structures, packing density, and branching may all contribute to the higher QA that we observed [91]. Furthermore, an animal study demonstrated that fluid leakage into the myelinated space due to the BBB permeability can also increase the diffusivity without actual white matter damage [92]. However, this association became non-significant after adjusting for potential confounds (i.e., age, sex, BMI, education, income, early life stress, medication status, current smoker, the severity of current symptoms of depression and anxiety, number of episodes, and the lifetime alcohol use). Therefore, caution is needed when interpreting these results. Both the fornix and thalamic radiations have been associated with decreased FA values in patients with MDD [66]. Future studies are needed to disentangle these contradictory results. While our study demonstrated a negative correlation between peripheral CRP concentrations and white matter integrity in multiple white matter tracts that are strongly implicated in depression, we did not find any significant correlation between QA values in those white matter tracts and depressive symptoms (as indexed by individual PHQ-9 items). One possibility is that the items of the PHQ-9 do not map well onto white matter pathways. Another possibility is that our sample size was not sufficiently powered to detect small effect sizes, as our statistical model indicated weak inverse associations between QA values from the identified tracts and the PHQ-9 total score, as well as a few other items (i.e., estimated standard beta coefficient = −0.11–−0.12, see Table 2). Although longitudinal studies consistently reported the robust association between elevated CRP concentration and depressive symptoms, the effect sizes were generally small [22,24]. Consistent with this literature, the effect size of the association between CRP and QA in the current study was also small, suggesting that CRP or the reduction in the QA may contribute to the etiology of MDD but explain very limited variance of the depressive symptoms measured by conventional clinical scales.

Several limitations are worth mentioning. First, we did not have a sufficient sample size of healthy controls in our dataset. Previous longitudinal studies included 680 non-demented elderly subjects and reported no significant association between CRP and white matter integrity [93]. This suggested that the effect of systemic inflammation on white matter integrity is likely to be very small in healthy populations. Thus, it will require a relatively large sample size in each group to provide sufficient power to detect significant interactions between diagnosis and immune markers. To avoid false negative results due to an underpowered sample, we did not include healthy controls in the current study. Future studies with larger samples in healthy subjects are therefore needed to adequately address this question. Second, while diffusion-MRI-derived measures such as QA or FA are known to be sensitive indices of white matter microstructural changes, it is important to recognize that QA does not directly quantify the biological properties of white matter (e.g., axon myelin). It quantifies the density of water molecules diffusing in an axonal direction, and can be influenced by many other factors related to fiber architecture [47]. For this reason, it can be difficult to interpret differences in QA values. Nevertheless, diffusion MRI is the only currently available method for non-invasively reconstructing white matter tracts and quantifying their structural integrity in the human brain. Our findings provide MRI-based evidence of peripheral inflammation effects on white matter integrity, which pave the way for further investigation into its underlying mechanisms. A third limitation is that not all proinflammatory cytokines were measured in this study. Although CRP is known to be correlated with proinflammatory cytokines such as IL-6, IL-1β, and TNF [17,18], other unmeasured markers (i.e., IL-6, TNF) may provide additional information about inflammatory status. Lastly, findings from our current cross-sectional study are associational and may be susceptible to measured or unmeasured confounds. We excluded subjects with significant or unstable medical conditions (i.e., cardiac vascular, gastrointestinal, endocrine, neurological, hematological, rheumatological, metabolic disorders, and autoimmune disorders), but participants with mild conditions such as hypertension, hypothyroidism, or taking nonsteroidal anti-inflammatory drugs may be included and could potentially influence CRP concentration and QA value. Longitudinal studies will therefore be important for validating the potentially causal links between inflammation and white matter microstructural integrity by examining changes over time following experimental manipulations.

## 5. Conclusions

As hypothesized, we found that elevated CRP levels were associated with decreased QA values within numerous white matter tracts connecting corticolimbic brain regions. This association remained robust after controlling for up to twelve possible confounds. While further research is needed to validate these associations, our findings support the neuroinflammation hypothesis of depression and shed new light on the negative influence of peripheral inflammation on the microstructural integrity of white matter pathways.

## Figures and Tables

**Table 1:** Demographic and behavioral data of the participant sample (*n* = 176).

	Mean	SD
Age	34.00	10.68
Sex (Male %)	33.50	-
BMI ^a^	28.86	5.22
Education ^b^	6.64	1.52
Income ^c^	9.76	2.89
CTQ ^d^	46.69	18.37
Medicated (%) ^e^	64.80	-
Current smoker (%)	13.00	-
Depression severity ^f^	61.62	7.17
Anxiety severity ^g^	62.32	6.69
Number of episodes ^h^	3.88	3.27
Alcohol use ^i^	4.99	2.54
Log CRP ^j^	0.53	1.44
**Depressive symptoms ^k^**	**Mean**	**SD**
PHQ-9 total score	13.16	4.94
Anhedonia	1.58	0.84
Depressed mood	1.53	0.82
Sleep problems	2.07	0.94
Tiredness	2.10	0.86
Changes in appetite	1.51	1.05
Feelings of inadequacy	1.79	1.00
Concentration problems	1.43	1.00
Psychomotor changes	0.72	0.83
Suicidality	0.44	0.69
**Ethnicity**	**%**	
Asian	1.14	-
Black	9.71	-
Hispanic	4.57	-
Native American	15.43	-
White	65.71	-
Other	3.43	-

^a^ BMI = body mass index; ^b^ measured by ordered categories. ^c^ Household income (log-transformed). ^d^ Childhood trauma questionnaire total score was used. ^e^ Medicated is defined as patients with MDD taking psychotropic medication. ^f^ PROMIS depression T score was used. ^g^ PROMIS anxiety T score was used. ^h^ Measured by MINI interview. Participants with over 10 episodes were treated as having had 10 episodes. ^i^ Log-transformed lifetime alcohol usage was used. Data obtained from CDDR interview. ^j^ CRP concentration (mg/L, log-transformed). ^k^ Measured by patient health questionnaire (PHQ-9).

**Table 2:** Associations between QA and depressive symptoms.

	QA Extracted from Blue Tracts ^a^			QA Extracted from Red Tracts ^b^		
Symptoms	SBC	95%CI	*p_uncorrected_*	SBC	95%CI	*p_uncorrected_*
Anhedonia	0.00	−0.15–0.16	0.96	−0.06	−0.21–0.10	0.48
Depressed mood	−0.01	−0.17–0.14	0.85	−0.01	−0.17–0.14	0.87
Sleep problems	−0.05	−0.21–0.11	0.52	−0.07	−0.23–0.08	0.36
Tiredness	−0.11	−0.27–0.05	0.19	−0.05	−0.20–0.11	0.55
Changes in appetite	−0.11	−0.26–0.05	0.20	−0.11	−0.27–0.04	0.16
Feelings of inadequacy	−0.06	−0.22–0.10	0.48	0.05	−0.10–0.21	0.51
Concentration problems	−0.12	−0.27–0.04	0.14	−0.01	−0.16–0.14	0.89
Psychomotor changes	−0.09	−0.25–0.06	0.25	0.03	−0.13–0.18	0.72
Suicidality	−0.04	−0.20–0.12	0.66	0.04	−0.11–0.20	0.59
PHQ-9 total score	−0.11	−0.27–0.05	0.18	−0.04	−0.19–0.12	0.63

^a^ Tracts showed a negative association with CRP concentration which was colored in blue for visualization in Figure 2A. ^b^ Tracts showed a positive association with CRP concentration which was colored in red for visualization in Figure 2A.

## Data Availability

The full preprocessing scripts and the statistical analysis R scripts used for current study are available upon request to the corresponding author.
